# Notes on Cholera and Its Treatment

**Published:** 1883-09

**Authors:** C. A. Gordon

**Affiliations:** Honorary Physician to Her Majesty the Queen


NOTES ON CHOLERA AND ITS TREATMENT. By Surgeon-General C. A. GORDON, M.D., C. B., Honorary Physician to Her Majesty the Queen.

Diarrhœa may occur as an independent affection in times of cholera, or it may happen as an early stage of the latter disease. When cholera prevails it is difficult to decide correctly and with precision where diarrhœa as such ends, and where distinctive cholera begins. The symptoms of the two affections differ in degree rather than in kind; but it is usually considered that the retention of color in and slower progress of diarrhœa by itself are to be taken as indicative of the milder malady.

Diarrhœa, when it occurs as a premonitory stage of cholera, has a duration seldom exceeding three days. As regards the prevalence of this affection among masses of population, it has been observed to vary from a few days to several weeks. In 1866 statistics regarding this point were taken in London. In 41 examples on that occasion recorded, the duration of the diarrhœa in 3 cases was 12 hours, in 1 case 18 hours, in 1 case 19 hours, in 7 cases 33 hours, in 12 cases 2 days, in 6 cases 3 days, in 2 cases 4 days, in 2 cases 5 days, in 1 case 6 days, in 1 case 7 days, in 2 cases 2 weeks, in 1 case 5 weeks, in 1 case 8 weeks. The general result of these observations shows that in more than half the number of cases diarrhœa preceded the more marked symptoms by less than three days. On that occasion in London there was a subsidence of diarrhœa before the outbreak of chol-era. A similar subsidence had preceded the outbreak of 1848-9 and 1853-4.

In some instances epidemics of diarrhœa have been considered to be really epidemics of cholera, although occurring in that form. In 1836 this was the case in the Indo-Chinese fleet. On that occasion the epidemic of diarrhœa which prevailed was considered to take the place of cholera which had in previous years occurred in the same fleet. At Huddersfield in 1849, diarrhœa and cholera prevailed simultaneously in so defined a manner that the cause which produced them both was considered to be identical, only the form of resulting disease to differ in different individuals. At Oxford in 1852, deaths took place by diarrhœa without the cases passing into confirmed cholera. And similar occurrences are noticed in other epidemics of the disease.

Persons coming from infected localities have been attacked with diarrhœa within a period extending to fourteen days from the date of their departure, the disease in them undergoing no farther development; and yet confirmed cholera occurred in the locality into which such persons had entered. Occurrences of this nature happened in France during the epidemic of 1855—notably at St. Germain, Melissy, St. Barthèlemy, and Villedieu. The Constantinople Commission acknowledged that cholera could be propagated in this manner.

It has come to be acknowledged as an axiom that the more severe the impending epidemic of cholera the shorter is the period of premonitory diarrhœa. In young persons diarrhœa in connection with cholera is considered to be of more frequent occurrence than it is in those more advanced in years. In a number of instances cholera has manifested a partiality to individuals habitually subject to diarrhœa.

Diarrhœa is said to occur more frequently in attendants on cholera patients, and in them to take the place of the more developed disease. In 1873 this circumstance was noticed in connection with the epidemic at Ottawa.

But although diarrhœa often precedes cholera, this does not happen invariably. There was no premonitory diarrhœa on the occasion of the cholera outbreak in the 86th Regiment at Kurrachee in 1846; none in the Madras epidemic of 1848. In the French epidemic of 1855 diarrhœa is mentioued by 97 reporters; by 15 its occurrence is considered exceptional. In the Crimea in that year our troops were seized, in many instances, with collapse of cholera without any premonitory symptoms whatever. At Gateshead in the same year 55 persons who went to bed in perfect health on the 25th of December, were attacked with cholera before sunrise on the 26th; and of the number so attacked, 32 were dead before sunset. In 1861 there was no marked prevalence of diarrhœa before or during the epidemic of cholera at Delhi. In 1866 diarrhœa, and indeed all other premonitory symptoms, were absent on the occasion of the cholera outbreak at Mean Meer. At Peshawur the attack of cholera in the 42d Highlanders was sudden, and unpreceded by premonitory symptoms. Other instances could be given in illustration of the fact that, although on occasions cholera is preceded by diarrhœa, this is by no means an invariable occurrence.

The success of the treatment applied in cases of cholera depends in a very great degree upon the early use of that treatment. For example, in London in 1849, there were treated 43,737 cases of "premonitory" diarrhœa, and of this number 58 only developed into cholera. In that year throughout England there were treated 130,000 cases of diarrhœa, of which number only 250 passed into cholera. A question was raised as to whether many of the cases so treated were not cases of simple diarrhœa. Perhaps they were; it is impossible to say. From calculations made in Glasgow in reference to the epidemic of that year, it has been stated that of persons treated within the first six hours after attack by cholera 21 per cent. died; of those first treated between six and twelve hours after attack, 33 per cent.; of those between the twelfth and twenty-fourth, 45 per cent.; and of those treated later, 66 per cent. In India the importance of early treatment is universally recognized, and special orders on this point are enforced in respect to soldiers. In 1877 the observance of such orders was followed by important success at Madras.

Briefly, the remedies administered in the stage of premonitory diarrhœa comprise carminatives, antispasmodics and astringents.

In the treatment of fully-developed cholera remedies of the most diverse nature have been used from time to time. Perhaps the earliest method on record was that which of late years has been called the "eliminative." That method dates from the year 360.

In the sixteenth century venesection, ligatures to the head, limbs and loins, acupuncture, pepper in rice-water as a drink and applied externally, were the means employed. After the violence of the disease had passed, purgatives were given.

In the seventeenth century the natives of India withheld liquids from patients affected with cholera, and cauterized the soles of their feet. Among the remedies administered were diffusible stimuli, saffron, opium, vegetable astringents, and ammonia. Towards the end of that century, and in the early part of the eighteenth, the French Missionaires at Trichinopoly administered to the cholera patients "un peu d'eau bénite et se mit à réciter quelques prières," with the happy result, as stated by themselves, that "le malade guérit subitement."

Among the remedies used in the eighteenth century were the following:—Calumba root in 1756; cassia fistula and rhubarb in 1761-63; opium and rice-water in 1769; Glauber salts with tartrate of antimony, in doses of one-eighth of a grain, in 1782; Madeira wine, laudanum and cordials also in that year; castor oil at Arcot in 1787; large doses of opium at Batavia in 1789.

During the portion of the nineteenth century already gone, methods of treatment in their nature absolutely the opposite of each other have been used. In 1817 large doses of calomel, combined with small quantities of opium, were given. In 1831 emetics of ipecacuanha were said to have "acted like a charm;" on the same occasion sulphur and phosphoric acid internally were given. In 1832 venesection, used, as already stated, in the sixteenth century, was reverted to; in 1849 the same method was for the third time practiced. Naphtha was employed for the disease as it occurred among the Prussian troops in the Caucasus; and, under the name of "elixir of Worenejo," that substance is believed to have been employed for the same purpose in Russia. In 1848-9 naphthaline and petroleum were given internally in the United Kingdom.

The use of opium vaunted by some writers is uterly condemned by others. During the epidemic in France, 1855, opium was extensively used; the result assigned to such use being that in some instances living persons were buried while in a state of narcotism only. There are many medical officers of great experience, who assert that it is chiefly among patients thus treated that the severe train of symptoms known as "secondary fever" occur.

With regard to ipecacuanha, about twenty reporters record their experience with that drug. Of 4,180 patients treated by means of large doses of it, 2,509 died.

Evacuants, praised by some writers, are by others stated to be deadly. The same remark applies to the use of tobacco fumes. Antispasmodics—notably, ether and chloroform—are used with the greatest advantage against spasmodic phenomena in the course of the disease.

Among the means applied externally and variously reputed have been frictions and excitants; hot baths, with and without aromatic herbs; bags of hot sand to the hands and feet; the application of cold; galvanism, etc., etc.

Among various other remedies used from time to time are the following, they being but a very few out of a long catalogue, namely:—Hot negus, with grated nutmeg; quinine, strychnine and nux vomica; sulphate of copper; homœopathy, practiced, it is said, during the epidemic in France with sad results; hypodermic injections of various kinds; inhalation of gases; transfusion of blood and of different saline mixtures; purges; astringents, carminatives; alkalies; sulphur; sulphuric acid; chloral, both by the mouth and hypodermically; acetate of lead; tartrate of antimony; stimulants; preparations of iron; hydrosulphuret of ammonia; olive oil; yeast; eupatorium, etc. For a time *Sumbul*, the product of Euryngium Sumbul, N. O.,

Umbellifeaæ, was used. In 1864 a solution of permanganate of potass was given in India.

In order to account for the insuccess of medical treatment in cases of declared cholera, the circumstance must be borne in mind that for the time being the function of absorption is in abeyance; especially is this the case in the algid stage of the disease.

But, while on the one hand there is no certainty of saving the life of a person attacked with cholera, neither is it right or justifiable to despair as to the results of means reasonably employed, even in cases the most desperate.—*Medical Press and Circular.*

